# Assessing the Spontaneous Spread of Climate-Adapted Woody Plants in an Extensively Maintained Collection Garden

**DOI:** 10.3390/plants12101989

**Published:** 2023-05-15

**Authors:** Krisztina Szabó, Attila Gergely, Barnabás Tóth, Kinga Szilágyi

**Affiliations:** 1 Institute of Landscape Architecture, Urban Planning and Garden Art, Hungarian University of Agriculture and Life Science (MATE), 1118 Budapest, Hungary; 2 Doctoral School of Landscape Architecture and Landscape Ecology, Hungarian University of Agriculture and Life Science (MATE), 1118 Budapest, Hungary

**Keywords:** woody plants, spontaneous spread, weed, invasive species, native plants, climate change, garden maintenance

## Abstract

Climate change may strongly modify the habitat conditions for many woody plant species. Some species could disappear from their natural habitats and become endangered, while others could adapt well to the changed environmental conditions and continue to survive successfully or even proliferate more easily. A similar process can occur within the artificial urban environment as the hitherto popularly planted urban trees may suffer from the extremities of the urban climate. However, among the planted taxa, there are species that spread spontaneously and appear as weeds in extensively managed gardens. In our study, we evaluated the native and non-native species involved in spontaneous spreading in the institutional garden of Buda Arboretum (Budapest) during the COVID-19 period in 2020–2021 when entry was prohibited, and maintenance went on in a restricted, minimal level. We investigated the correlation between spontaneously settling and planted individuals, and then performed multivariate analyses for native and non-native spreading plants for spatial and quantitative data. During our studies, we observed the spontaneous spreading of 114 woody species, of which 38 are native and 76 are non-native. Taking the total number of individuals into account, we found that, in addition to the 2653 woody species planted, a further 7087 spontaneously emerged weeds developed, which creates an additional task in the maintenance.

## 1. Introduction

The priority tasks for the maintenance of urban green spaces include the removal of spontaneous weeds or invasive species. Plants appearing in undesirable places and conditions may cause problems for residents and maintainers with their constant pressure on the existing vegetation. They can limit the growth, reduce the habitat, and result in amorphous, asymmetrical growth due to fight for light or reduced water or nutrient uptake in a divided root zone. Botanical and collection gardens are often leaders in the introduction of new plant species for public green or garden use; their recommendation, introduction, and commercialization require scientific soundness and professional responsibility [1,2,3,4].

Botanical gardens play an essential role in the observation and study of plants. Their role is unquestionable in research projects on climate change, phenological monitoring of plant adaptation strategies, and physiological processes [5,6,7]. These experiences can help with the planting design of public green spaces. When working on plant selection, we must know which species are acceptable and which plants should be avoided from an ecological aspect. Gardens often push the limits of species’ distribution [8]. Taxa with questionable survivorship may be problematic to maintain in public green spaces; still, others are easy to care for because they are stable and they thrive and reproduce, as reported in several studies [1,2,3,4]. A suitable planting design may significantly reduce the maintenance tasks.

Spontaneously occurring tree species in urban environments include native species such as *Acer campestre*, *A. platanoides*, and *Ulmus* spp., which have a spreading ability similar to invasive plants. These are referred to as ‘spreading native species’ [9,10]. In this case, the relevant literature [11,12] agrees that the term ‘invasion’ is not appropriate to describe the spread of native plant species, even when their population increases. Some non-native species belonging to the invasive lists are ready to ‘escape’ and spread from their original plantation and can pose significant ecological and economic challenges.

Several definitions of invasive species exist in the literature. According to Richardson et al. [11], an invasive species is defined as any non-native species introduced directly or indirectly into a region with an increasing population. Hilton-Taylor and Brackett’s definition takes the habitat consequences of a species into consideration, i.e., the damage it causes to semi-natural and natural habitats [13]. The spread of invasive species and the habitat conversion impacts due to human activities could result in habitat fragmentation and significant transformation [14]; the process may end in a severe loss of biodiversity, ecosystem degradation, and reduced resilience to the disturbance in both natural floras [15,16] and planned green spaces. Moreover, according to Pimentel et.al. (2005) [17], social attitudes and public perceptions often attribute, for example, damage to roads and building foundations to invasive plants, in addition to causing public health problems. However, the term weed can refer to native and non-native taxa, species that settle in valuable green areas and thus cause a high maintenance challenge.

In landscape architecture and horticultural practice, the adaptability and applicability of plant species with different conservation statuses (protected, highly protected, and invasive) are common issues [18]. Invasive species, which often start their careers in botanical gardens, have the opposite effect on conservation. Plants, once released from gardens, can infest large areas due to their high reproductive patterns and aggressive spread, causing severe ecological and economic damage. Control is often a problem, even in intensively managed plantations.

The 100 most dangerously invasive organisms (ISSG 2017) [19,20] include 21 woody species on a global scale. Some of these tree taxa (e.g., *Acacia mearnsii* De Wild, *Lantana camara* L., *Pinus pinaster* Aiton, *Rubus ellipticus* Sm., *Schinus terebinthifolius* Raddi., *Tamarix ramosissima* Ledeb., and *Ulex europaeus* L.) often occur in Europe, causing severe ecological problems. The same list compiled for Europe (on a continental scale) contains 16 woody species; 2 of them (*Ailanthus altissima* Mill. and *Robinia pseudoacacia* L.) belong to the invasive classification in Hungary [21,22,23].

Fortunately, most introduced species have spread only on a local scale, and they are under control. However, an insignificant minority of species have become self-sufficient and have spread beyond all imagination [18]. The spread of Indian and American poke (*Phytolacca acinose* Roxb. and *P. americana* L.) may cause severe ecological problems in the sandy habitats of Hungary (Transdanubia and the southwestern region); the false indigo (*Amorpha fruticosa* L.) and boxelder maple (*Acer negundo* L.) have spread all over in the floodplains. In drier areas and, unfortunately, even in the mountains, the black locust (*Robinia pseudoacacia* L.) and their peers are appearing ever-increasingly.

Botanical gardens have regularly collected information on alien species from the beginning [7,19]. According to many publications, botanical gardens and the horticultural trade seem to be the first steps for invasive species [1]. In 2013 the association of European Botanical Gardens adopted a standard code of conduct for the management of invasive species in gardens [24], which, for example, manifested itself in a decrease in the seed exchange of invasive species [25].

The university in the southeast region of Hungary (The University of Szeged, Department of Geoinformatics, Physical and Environmental Geography) has created a GIS database for the territorial distribution of the most common invasive plant species in Hungary [26]. The database shows the occupation of several invasive species in different years. For example, the first Hungarian Black List (invasive tree and shrub species) appeared in 2002 and based on observations, the latest one was published in 2020 [27]. This comprehensive technical publication discusses a collection of invasive species, the so-called Black List and the Grey List, which describe the potentially invasive species.

This research investigates the quantitative proportions and species composition of invasive (non-native species) (IAS) [28] and all other spontaneously spreading species and the relationships between their distribution and sustainability in an extensively maintained garden such as the Buda Arboretum. This study draws attention to the taxa that may have the potential to spread in public areas due to the changing climate. The field survey occurred during a special period in 2020–2021 when garden management had to remove the regular garden workers’ and the horticultural and landscape architect students’ participation in garden maintenance due to the strict COVID-19 closures and restriction.

Our research questions are as follows:

What are the relationships between the abundance and composition of spontaneously occurring species and individuals and the size of patches and green patches? For which species are there correlations between the number of individuals established and spontaneous occurrence? Are patterns and regularities of spreading observed for species? What is the proportion of invasive and aggressively spreading native species among the spontaneously occurring species? Which taxa may threaten the maintenance of green areas in public spaces? Are there any taxa that should be monitored for their potential to spread?

## 2. Results

### 2.1. Spontaneously Settled Species in the Study Area

During the Buda Arboretum site survey, we encountered a lot of weeds. The total plant collection consisted of 2108 taxa (basal species, varieties, forms, and variants). We found 114 taxa that can self-sustain and spontaneously appear in many parts of the garden, weeding out the intended concept and causing maintenance difficulties. The proportion of native and non-native plants was approximately one-third (38 taxa) to two-thirds (76 taxa) (Table 1). The number of spontaneously dispersed specimens was 7022, which represents an additional 265% of the plants planted in the garden compared to the arboretum (2653 specimens). The total number of spontaneously spread individuals was 7022, which represents a 265% extra load on the garden compared to the plants planned and planted in the arboretum (2653 individuals). Of these, 4186 individuals are non-native, which is almost 60%. The diversity calculations for the groups/spread types (see below) are presented in the following sections.

**Figure 1:**
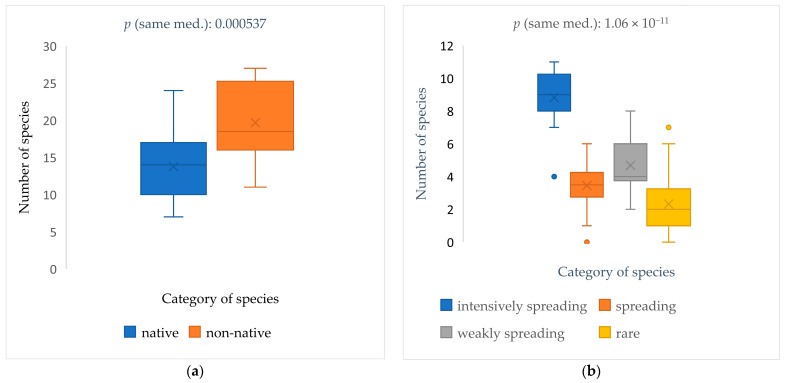
Boxplot of taxa number (species) in the plots (‘parcels’): mean, median, 25–75% quartiles, non-outlier range, and outliers. Mann–Whitney test for “equal medians” (**a**) and Kruskal–Wallis test for “equal medians” (**b**).

There is a significant difference between the sample medians in both case (Figure 1a,b). According to the results of the diversity analysis, there are significantly more non-native species present in the plots; there are also significantly more intensively spreading species in the plots (‘parcels’).

Based on the study of Bartha (2020) [27], we focused on the black-listed and the Grey-listed (the potentially invasive) taxa detected in the Buda Arboretum (Table 2 and Table 3). The invasive dendro-taxa showed a wide variety; unfortunately, some of these invasive plants are already active (directly or indirectly threatening the native species through habitat modification), while others are on the watch list but are not yet managed (Table 3). 

### 2.2. The Spontaneous Emergence Categories

#### 2.2.1. Category I

**Figure 2:**
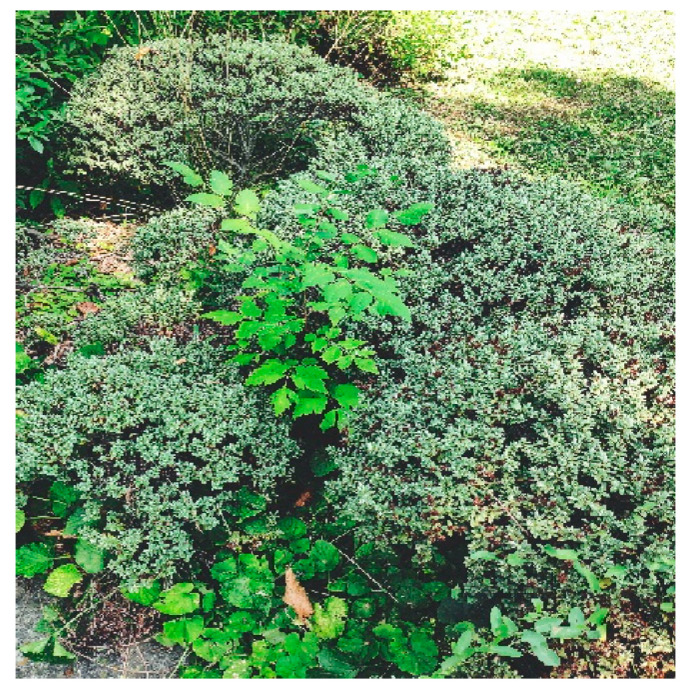
The common hackberry (*Celtis occidentalis* L.) in the patch of evergreen shrubby veronica (*Hebe pinguifolia* Cockayne & Allan) photo by Krisztina Sz.

**Figure 3:**
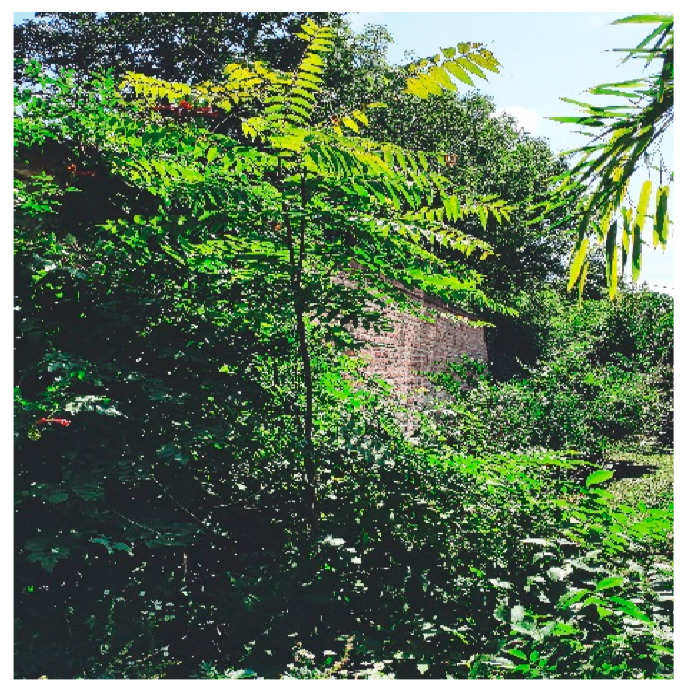
The tree of heaven (*Ailanthus altissima* Mill.) large seedling in the shrub in front of the wall, photo by Krisztina Sz.

**Figure 4:**
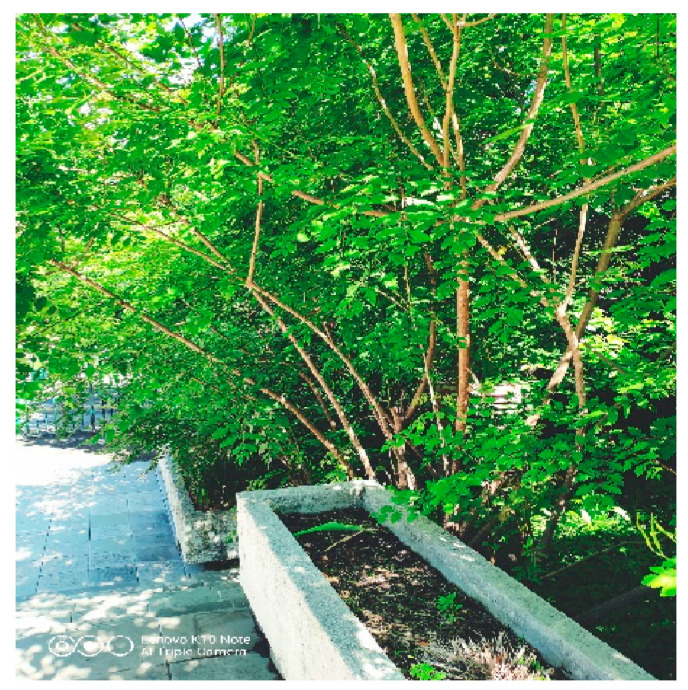
A weeding bug of goldenrain tree (*Koelreuteria paniculata* Laxm.), photo by Krisztina Sz.

Among the alien species, the spontaneous occurrence of **intensively spreading** species was recorded for 11 species or related species. In total, the 11 species on the 22 plots of the arboretum represent 2933 individuals; the common hackberry (*Celtis occidentalis* L.) recorded the highest number of 561 individuals. In contrast, the lowest number of individuals in this category belongs to the white mulberry (*Morus alba* L.). The intensively propagating species are represented by four black-listed taxa (*Ailanthus altissima* Mill, *Celtis occidentalis* L., *Parthenocissus* spp., and *Robinia* spp.). Among the operative taxa of the Grey List, we detected three taxa in the garden (*Mahonia* spp., *Morus alba* L., and *Prunus cerasifera* Ehrh.). The species for observation are represented by four taxa (*Cotoneaster* spp., *Diospyros* spp., *Koelreuteria paniculata* Laxm., and evergreen *Lonicera* taxa). Some examples are shown in Figure 2, Figure 3 and Figure 4. We made the numbering at the genus level for the American five-leaved creeper, holly, cotoneasters, persimmons, and evergreen honeysuckle. *Diospyros lotus* L. in 99% of persimmons, and three taxa of *Lonicera fragrantissima* Lindley & Paxton, *L. standishii* Carriére, and their hybrid, *L.* × *purpusii* Rehder are observed in evergreen honeysuckle.

Among the expanding native species, 38 different taxa have been recorded as spontaneous occurrences. Of the native species, the maple (Acer spp.) genus has an outstanding ability to spread. We found the maple (*Acer platanoides* L.) to be the winner. However, it is a negative victory, not only in the group of native taxa but also among all non-native invasive species in the Buda Arboretum. In addition to the native maple, even the sycamore maple (*Acer pseudoplatanus* L.) is represented among the intensively spreading (I. category) woody taxa. Two more woody taxa, the field maple (*Acer campestre* L.) and the linden (*Tilia* spp.), and five shrubs (the erect habit elderberry (*Sambucus nigra* L.), the common dogbane (*Cornus sanguinea* L.), the wild plum (*Ligustrum vulgare* L.), and roses, mainly dog rose (*Rosa canina* L.), and others such as creeping clematis (*Clematis vitalba* L.)), belong to the same category. Each of them pollutes the arboretum with more than 100 specimens, the maple with 647 specimens.

#### 2.2.2. Category II

For the **spreading** species, 522 individuals of 8 species were weeded in the arboretum, representing an average of 19% per unit area in this category. However, two of the Black List species, bush maple (*Acer negundo* L.) and ash (*Fraxinus* spp.), were only recorded at the genus level. Among the Grey List species, the wine raspberry (*Rubus phoenicolasius* Maxim.) represented a reasonable spread. The remaining taxa in this category were cock’s-foot (*Crataegus crus-galli* L.), bright holly (*Ligustrum lucidum* W.T.Aiton.), Korean holly (*Ligustrum ovalifolium* Hassk.), cat-root (*Smilax excelsa* Duhamel), and viburnum (*Viburnum* spp.). Perhaps the surprise species among these was the evergreen-leaved glossy privet from (Latin) East Asia. No specimens of this category were found in plot 21.

Among the expanding native species in the spreading category, there are four taxa, from which we examined the manna ash (*Fraxinus ornus* L.) as exact species, while the others are listed as a genus, oaks (*Quercus* spp.), viburnums (*Viburnum* spp.), and elms (*Ulmus* spp.).

#### 2.2.3. Category III

In the category of **weakly spreading**, 23 different taxa with a total of 587 individuals were observed with the spontaneous appearance of 15%/m^2^ on average: horse chestnut (*Aesculus hippocastanum* L.), Chinese barberry (*Berberis julianae* C.K.Schneid.), trumpet creeper (*Campsis* spp.), Judas tree (*Cercis siliquastrum* L.), American yellowwood (*Cladrastis kentukea* (Dum. Cours) Rudd.), fontanesia (*Fontanesia phillyreoides* Labill.), Kentucky coffeetree (*Gymnocladus dioicus* (L.) K.Koch.), Japanese honeysuckle (*Lonicera japonica* Thunb.), Tatarian honeysuckle (*Lonicera tatarica* L.), wolfberry (*Lycium barbarum* L.), Persian ironwood (*Parrotia persica* C.A. Mey.), poplars (*Populus* spp.), prunus (*Prunus* spp.), pagoda tree (*Styphnolobium japonicum* syn. *Sophora japonica* L.), common lilac (*Syringa vulgaris* L.), bee-bee tree (*Tetradium daniellii* (Benn.) T.G.Hartley), Chinese cedar (*Toona sinensis* M.Roem.), grapes (*Vitis* spp.), and Chinese wisteria (*Wisteria sinensis* (Sims) DC.). Three black-listed species from the categories wolfberry, common lilac, and Chinese cedar, as well as operational Grey-listed taxa poplars and prunus. Of the others, more attention should be paid to the maintenance of Chinese barberry, trumpet creeper, Judas tree, poplars, and Chinese wisteria, which are more common in urban applications.

Among those indigenous taxa, appearing with less than 50 individuals, includes species such as Tatarian maple (*Acer tataricum* L.), common hawthorn (*Crataegus monogyna* Jacq.), common ash (*Fraxinus excelsior* L.), Tatarian honeysuckle (*Lonicera tatarica* L.), poplars (*Populus* spp.), sweet and bird cherry (*Prunus avium* L., *Prunus padus* L.), buckthorn (*Rhamnus cathartica* L.), silver linden (*Tilia tomentosa* Moench), and common hazel (*Corylus avellana* L.).

#### 2.2.4. Category IV

To the infrequent, **just emerging** category belong 30 taxa and 107 individuals representing an average of 4%/m^2^ load, including black-listed chocolate vine (*Akebia quinata* (Thunb. ex Houtt.) Decne.), together with the evergreen taxa of the genus level silverberry (*Elaeagnus* spp.) and common hoptree (*Ptelea trifoliata* L.). Grey-listed species include paper mulberry (*Broussonetia papyrifera* L), summer lilac (*Buddleja davidii* Franch.), Mediterranean hackberry (*Celtis australis* L.), honey locust (*Gleditsia triacanthos* L.), and sumac (*Rhus* spp.). The spontaneous appearance of some warm-demanding species, Jerusalem thorn (*Paliurus spina-christi* Mill.), holly oak (*Quercus ilex* L.), English holly (*Ilex aquifolium* L.), and Chinese-pepper (*Zanthoxylum simulans* Hance), has been surprising; in some cases, the evergreen oaks grow hundreds of meters away from the mother plant.

Interestingly, among the expanding native species, the spontaneous spreading of smoke trees (*Cotinus coggygria* Scop.) is not significant from the emerging category; however, much of the garden is non-irrigated, and sunny places on a southern slope would be suitable. Among other species, the edible fruits are worth interest, such as snowy mespilus (*Amelanchier ovalis* Medik.), simple barberry (*Berberis vulgaris* L.), wild pear (*Pyrus pyraster* L. Burgsd.), and Turkish hazel (*Corylus colurna* L.). Interestingly, we found European beech (*Fagus sylvatica* L.) seedlings in the sunny and warm part of the garden.

#### 2.2.5. Category V

Among the vegetative propagating and **colonies forming**, some taxa may cause significant maintenance surplus, such as, e.g., Caucasian spurge (*Andranchne colchica* Fisch & C.A. Mey. ex Boiss.), Japanese knotweed (*Fallopia japonica* Houtt.), eastern tree (*Forsythia* spp.), sumac (*Rhus*), alpine currant (*Ribes alpinum* L.), and bamboos, of which the rapidly spreading, oppressive colonies of Japanese knotweed and bamboos should be given priority in the future.

The number of native plants prone to colony formation is only two. One is the Russian almond (*Prunus tenella* Batsch.), planted in two places in the lower garden. The plant propagates by root suckers, hence the current patch size is several times larger than the original planting. The spreading colony thus threatens the survival of the surrounding plantation and reduces their ornamental value. The other is the common ivy (*Hedera helix* L.), which also has several adult individuals and can spread by vegetative creeping and rooting shoots or generatively too. In many cases, the vigorous shoots of ivy climb up into the crown and create a separate ‘crown of ivy’, which can be harmful and even dangerous to the supporting parent plants in a short future.

**Figure 5:**
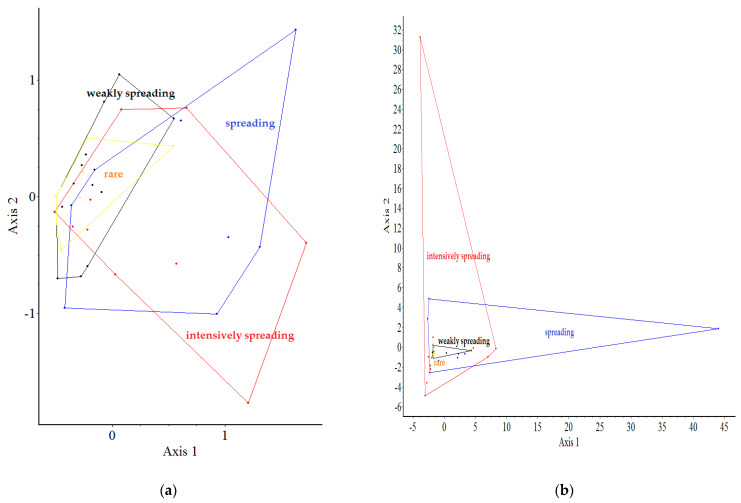
Partition (groups of spreading types) superimposed on ordination (PCoA) for spontaneously spreading species occurrences as object data. In case (**a**) binary, the eigenvalues of the 1st and 2nd axes were 14% and 13%, respectively, and in case (**b**) quantitative, the eigenvalues of the 1st and 2nd axes were 33% and 16%, respectively. Legends: red—intensively spreading (Cat. I); blue—spreading (Cat. II); black—weakly spreading (Cat. III); yellow—rare/just emerging (Cat. IV).

Figure 5 shows the results of our analysis on the connection between the spontaneously spreading species groups. In terms of the four groups (Cat. I–IV) in the binary data, the results show that there are two relatively distinct groups: intensively spreading (Cat. I) and spreading (Cat. II), but the others are very similar. For all these quantitative data, the objects (species groups) are “similar” to each other, and the groups are overlapping, apart from the two “outlying” species (*Robinia* spp. and *Rubus phoenicolasius*).

### 2.3. Correlation between Spontaneously Emerging and Established Individuals Per Plot

Almost all the correlations gave positive results, and some species are strongly correlated. Among the non-indigenous *Disopyros* species, *Prunus cerasifera* are in the group of intensive spreaders in Cat. I, *Fraxinus* species in Cat. II, and *Fontanesia phillyreoides*, *Gymnocladus dioicus*, and *Lonicera japonica* in Cat. III. *Toona sinensis* and *Wisteria sinensis* show a strong positive correlation. Among the native species, *Acer pseudoplatanus*, *A. tataricum* and *Ulmus* species show a strong positive correlation (Table 4). In terms of correlation, only some creeping, vine-like, and strongly rooted species showed a negative correlation, for example, *Rubus* spp., *Campsis* spp., *Clematis vitalba*, and *Lycium barbarum*.

### 2.4. Effect of the Area on the Number of Individuals

**Figure 6:**
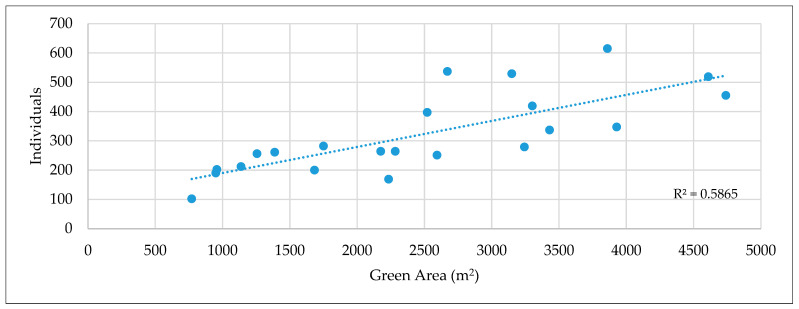
Linear regression of spontaneous spreading species in plots (I–XXII parcels).

In the regression analysis for the plot-by-plot assessment of the relationship between green space (area) and spontaneous species (total number of individuals), the regression coefficient is R^2^ = 0.5865, i.e., the size of the green space explains 59% of the variation (abundance) of individual species in each plot (Figure 6).

### 2.5. Comparison of Plots Using a Multivariate Analysis

**Figure 7:**
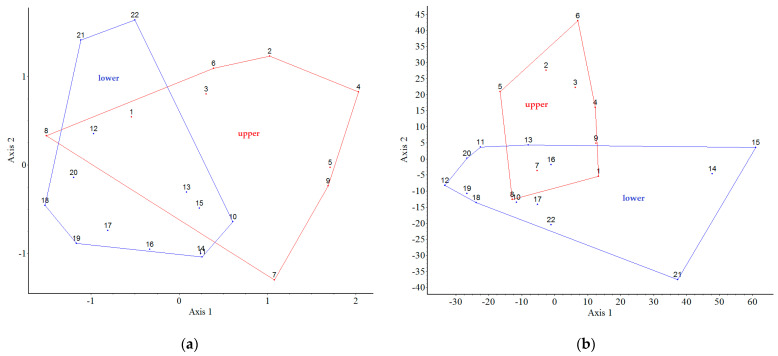
Partition (groups of the plots of upper and lower garden) superimposed on ordination (PCoA) for the native species occurrences as object data. In case (**a**) binary, where eigenvalues of the 1st and 2nd axes were 19% and 13%, respectively. In case (**b**) quantitative, where eigenvalues of the 1st and 2nd axes were 36% and 20%, respectively. In both cases, the red cluster indicates the upper garden, and the blue one indicates the lower.

Figure 7 shows the results of our analysis on the connection between the native species and plots. There are no distinct plots based on the presence of native species (38 species), but there are very similar plots (clusters), e.g., 11-14, 5-9, which are not adjacent. However, for the binary data (species presence or absence) the lower and upper gardens are observed to be distinct (Figure 7a). In terms of the quantitative data (individuals of species in plots), distinct (similar) plots can be observed: 14-15-21 and six are self-contained. The other plots are “similar” to each other; they are in a cluster (Figure 7b).

**Figure 8:**
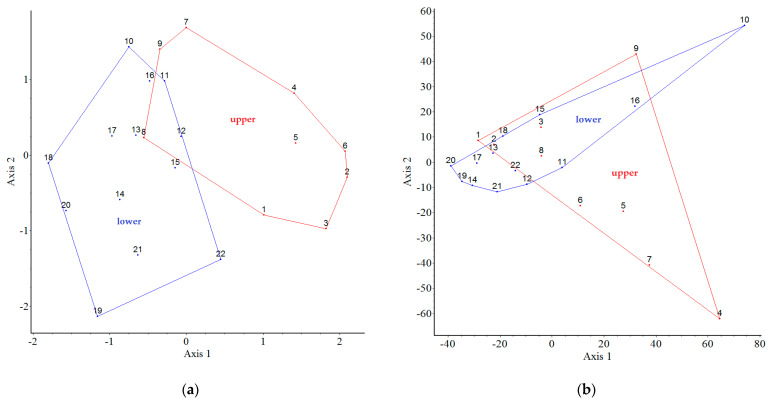
Partition (groups of the plots of upper and lower garden) superimposed on ordination (PCoA) for the non-native species occurrences as object data. In case (**a**) binary, where eigenvalues of the 1st and 2nd axes were 14% and 10%, respectively. In case (**b**) quantitative, where eigenvalues of the 1st and 2nd axes were 31% and 19%, respectively. In both cases, the red cluster indicates the upper garden, and the blue indicates one the lower.

Figure 8 shows the results of our analysis on the connection between the non-native species and plots. In terms of the presence of non-native taxa (76 species) in the binary data, the results show that there are no distinct groups (plots), but there are very similar ones, e.g., 8-13, 11-16 (Figure 8a). For all these quantitative data, the non-native invasive taxa have distinct plots (clusters), e.g., 4, 5-6-7 and 9-10-16. The other plots are “similar” to each other; they are in the same big cluster (Figure 8b).

**Figure 9:**
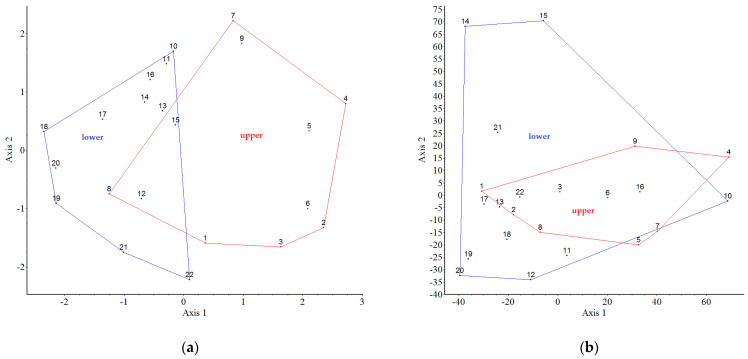
Partition (groups of the plots of upper and lower garden) superimposed on ordination (PCoA) for the native and non-native species together occurrences as object data. In case (**a**) binary, where eigenvalues of the 1st and 2nd axes were 14% and 11%, respectively. In case (**b**) quantitative, where eigenvalues of the 1st and 2nd axes were 23% and 15%, respectively. In both cases, the red cluster indicates the upper garden, and the blue one indicates the lower.

Figure 9 shows the results of our analysis of the connection between all spontaneously emerging/spreading species (native species and non-native together) and plots. If we compare the native and non-native species together, i.e., the species assessed by the spontaneous occurrence, then, when applied to the binary data (presence or absence), we find that the lower garden is distinct from the upper garden, within which the adjacent ones are similar (Figure 9a). If we compare the native and non-native species together, i.e., the species assessed by the spontaneous occurrence, then, when applied to the quantitative data (individuals), we find that the lower garden is not distinct from the upper garden, but some adjacent plots (parcels) are very similar (clusters): 2-3-6, 4-5, 7-9, and 10-11-13-14-15-16 (Figure 9b).

Our results—the partition superimposed on the PCoA ordinations shows clear differences between the upper and lower garden—confirm that the position of the plots (parcels) has an impact on both native and non-native woody plants.

## 3. Discussion

### 3.1. Woody Weeds in the Garden and Other Habitats

In addition to the well-known woody weeds, especially in extensive areas, such as *Ailanthus altissima* Mill, *Acer negundo* L., which is very widespread in Hungary, *Celtis occidentalis* L., *Koelreuteria paniculata* Laxm., *Ulmus pumila* L., and *Prunus cerasifera* Ehrh. Our arboretum research could not confirm the increasing weeding ability of *Celtis australis* L., though the invasion problem seems relevant in the southern regions of Hungary [29,30]. We have measured many specimens not only in the arboretum but in other extensively managed areas, such as the *Acer platanoides* L. seedlings in these unfavorable urban open spaces. Similar problems occur with *Acer campestre* L. and *Fraxinus excelsior* L. species. The spontaneous occurrence of *Cornus sanguinea* L. was very high in the botanical garden, though, as this species is rarely used in public spaces, we did not encounter its firm weed control. Due to its high environmental tolerance, its vast and expanding patches are often in nature with species from the same environmental conditions as, for example, *Prunus spinosa* L. on the southern slope of Bükk mountain (in the northeast region of Hungary) and other natural areas [31]. High numbers of *Sambucus nigra* L. were recorded in the garden, a weed in public areas, especially in neglected areas. [31]. High numbers of *Sambucus nigra* L. were also recorded in the garden, a weed in public areas, especially in neglected areas. Spontaneously emerging individuals of *Ailanthus altissima* Mill, *Celtis occidentalis* L., *Acer platanoides* L., *Acer pseudoplatanus* L., *Cornus sanguinea* L., *Clematis vitalba* L., *Koelreuteria paniculata* Laxm., *Ligustrum vulgare* L., *Mahonia aquifolium* (Pursh) Nutt., *Morus alba* L., *Parthenocissus* spp., *Prunus cerasifera* Ehrh., *Robinia* spp., *Rosa* spp., and *Sambucus nigra* L. species occurred in almost all sites (plots), regardless of where they were planted; hence, they are invasive plants because their seeds can cover large areas and cause weed infestation in remote areas for kilometers from the parent plant. Their dominance is a huge problem among the individuals presented. In semi-natural, extensively maintained public areas, ineradicable colonies of *Poligonum japonicum* (Houtt.) Ronse Decr. may threaten habitat planting.

### 3.2. Potential Weeds in Public Open Spaces

The results of the arboretum survey draw attention to the spread or potential proliferation of several species that are still not listed in the conservation protocols. In most cases, these potentially weeding plants grow in private gardens and, though rarely, in public open spaces. It seems that their capacity for rapid acclimatization and high tolerance will cause maintenance difficulties in the future, even in extensively managed areas. The first to be discussed here are the evergreen honeysuckles, which belong to the taxa *Lonicera fragrantissima* Lindl. & Paxton, *L. standishii* Carriére and their hybrid *Lonicera* × *purpusii* Rehder, already mentioned. All three taxa are decorative plants with showy morphological features all year round. They are true winter garden plants, growing quickly and with a rich and fragrant flowering in early spring; furthermore, they often bloom in winter and are prone to second flowering (remontage). Their tasty, sweet, fleshy, and red-ripening fruits are suitable for bird feeding, though birds can be responsible for the seeds’ distribution. In the planting design, these plants are suitable for space forming, as they can form groups, patches, and appear solitary too.

Of the genus *Diospyros*, the *Diospyros lotus* L. is able to spontaneously emerge. The fruits ripen well in our region, and their seeds germinate and strengthen far from the mother plant. There is a spontaneous sprouting specimen in the Buda botanical garden that has grown into a solitary tree over the past few decades. Since it was a plant rarity about 30–50 years ago, gardeners left it. Some *Diospyros kaki* L. seedlings also live in the arboretum, though their numbers are not significant as their fruits are difficult to ripen in the garden.

Another unique species is *Ligustrum lucidum* W.T.Aiton, which is present in the Buda Arboretum in significant numbers. It can be an excellent solitary large shrub or tree with its evergreen, large, and spectacular leaves. With its fragrant flowers and long-lasting bluish and black fruits, it could be a popular ornamental plant, but its potential ‘release’ needs attention. Nevertheless, according to our phenological observations, it well-tolerated the extremely dry weather in 2021 (the driest year in an 80 years’ period).

### 3.3. Proposal for Reclassification: A Prelude to Change

Based on our research, some potentially invasive species [27] need more attention in the future; so we propose to move them from the watch list to the operational list. Examples include *Cotoneaster* spp., *Diospyros lotus* L., *Koelreuteria paniculata* Laxm., and evergreen *Lonicera* taxa. We have recorded more than 250 individuals of *Koelreuteria paniculata* Laxm., and our observations show that it causes weed infestations and maintenance problems, not only in the arboretum but also in public spaces. It is one of the most common plants in residential green areas, along with *Acer platanoides* L., *Acer negundo* L., *Ulmus* spp., and *Ailanthus altissima* Mill. The spontaneous spread of *Gleditsia* ub the observation category is not significant in the arboretum because there are thornless (f. *inermis* L.), rather cultured species without fruits. However, our previous site analyses found that the basic species are all along roadsides, in old castle gardens, historic gardens [32], and even in urban green spaces. The *Celtis australis* L. is likely to cause problems in public areas in the future, similarly to its relative, the *Celtis occidentalis* L. However, the 2021 surveys did not confirm this.

The local distribution observed in *Pterocarya fraxinifolia* (Poir.) Spach., *Rhus* spp., *Fallopia baldshuanica* (Regel) Holub., *Rubus phoenicolasius* Maxim., *Juglans regia* L., *Rosa* spp., and *Robinia* spp. taxa occurred in 80–90% of the plots with variable individuals. The evergreen *Lonicera* spp. and *Diospyros lotus* L. are present in the arboretum in high numbers, though they are not common yet in public use.

Of the operational Grey List taxa found in the arboretum, *Buddleja davidii* Franch., *Paulownia tomentosa* (Thunb.) Steud., *Populus* spp., and *Prunus mahaleb* L. are rare, while *Mahonia* spp., *Morus alba* L., *Parthenocissus* spp., and *Prunus cerasifera* Ehrh. are common. *Phyllostachys viridiglaucescens* (Carriére) Riviére & C. Riviére shows a local spread, but presents a relevant biotope problem for the neighboring plants.

### 3.4. Spontaneous Spreading Abilities of Several Planted Individuals

**Figure 10:**
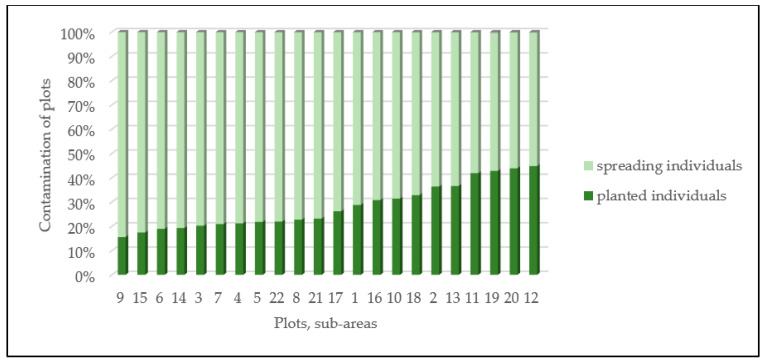
Contamination of garden plots: spontaneously spreading and invasive individuals in the Buda Arboretum.

The survey may help the garden management plan with the timing and sequencing of maintenance works and defining the urgent interventions. The number of spreading specimens detected in the survey clearly shows the most neglected parts of the Buda Arboretum (Figure 10).

Recommendations for public open spaces should also consider how spontaneous emergence evolves concerning the numbers planted. A few examples highlighted within the survey results prove (Table 5) that the number of planted specimens seems to be the determining factor in spontaneous spreading, and hence, the number of invaded specimens. The table shows that the ratio of spontaneous and planted individuals is a preferable indication of plants’ spreading or their spontaneous emergence capacity, even in public application practices. According to these rates, there is no relevant difference between the native taxa *Acer platanoides* and *Acer pseudoplatanus*. The most problematic non-native species are *Celtis occidentalis*, *Ailanthus altissima*, *Koelreuteria paniculate,* and *Diospyros lotus*.

There is only a partial agreement between the results in Table 4 and Table 5. According to the correlation per quadrat (per plot) among the highly correlated (r ≥ 0.7) ones, only *Prunus cerasifera* and *Diospyros* spp. belong to the individuals planted and spontaneously spread in the total green area. This may suggest that the taxa of the highly correlated assemblages (Table 4), except for the *Diospyros* and *Prunus* taxa, are typical in their spontaneous occurrence in the vicinity of the planted parent plant, especially in the case of the weakly spreading (Cat. III) species (e.g., *Wisteria* spp., *Fontanesia phillyreoides*, *Gymnocladus dioicus*, *Lonicera japonica*, and *Acer tataricum*).

### 3.5. Diversity Analysis and Other Additional Correlations

According to the results of the diversity analysis, the plots have significantly more intensively spreading alien species due to their successful dispersal, lack of pests, and the reduced competition that they face in their new habitats [10].

The PCoA assignments based on the data collected show clear differences between the upper and lower gardens—confirming that the location of the garden plots has an impact on both native and non-native woody plants. The relationship between the groups of spontaneously dispersing species, based on the four binary data sets (Cat. I–IV) forms two relatively distinct groups: intensively dispersing (Cat. I) and dispersing (Cat. II), while the others are similar. For all these quantitative data, the objects (species groups) are “similar” to each other, with the groups overlapping except for the two “outliers”.

Our results confirm that the size of a green space has a moderate effect on the total number of native and non-native woody plants’ individuals.

## 4. Materials and Methods

### 4.1. Research Area—Buda Arboretum

**Figure 11:**
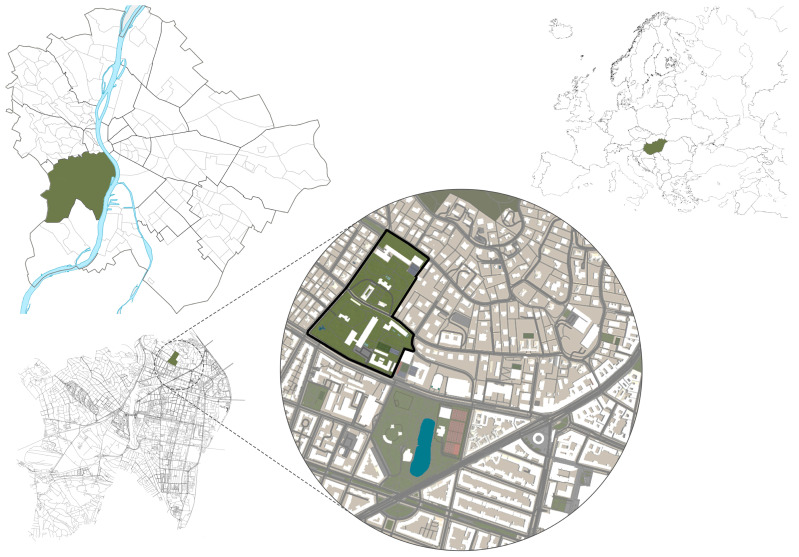
Location of the Buda Arboretum (illustrations by Barnabás Tóth).

“Green island or green oasis in the heart of the bustling city”—that is what you can read about the Buda Arboretum in Budapest (Figure 11) [33]. The Buda Arboretum, one of the richest ones in Hungary, was officially founded in 1894 [34,35], and it is a nature conservation area of metropolitan status (1975). It plays a vital role in the life of the district and the green space system of Budapest as a valuable ecological, conditioning, and cultural–educational area.

**Figure 12:**
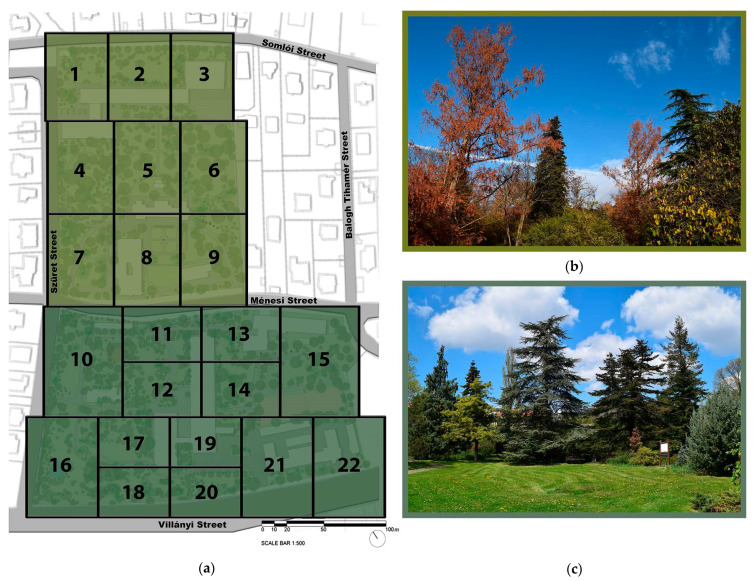
(**a**) The upper (in light green) and the lower gardens (dark green) with their 22 sub-areas; two characteristic photos of the Buda Arboretum; (**b**) upper garden and (**c**) lower garden (illustrations and photos by Barnabás Tóth).

The 7.5 hectares-large arboretum is situated on the southern slope of Gellért Hill and is divided by the Ménesi Street into two parts, the so-called lower and upper garden. The upper garden part is divided into the university library, the dormitory, and the sports hall, which, like the Ménesi Street, create a significant ecological barrier between the two gardens [36,37,38]. The entire garden area consists of 22 plots, with a varied green space and planted stock shown in a geometric pattern of Figure 12.

The planting of ornamental plants in the upper garden began in 1893, designed by Károly Räde, the chief gardener of the Horticultural Academy, who was responsible for garden development [36,37,39,40]. The plant population has been constantly developing and changing [41]. Our database shows precisely that the garden contains 2108 different tree taxa with 2653 planted specimens. There is an ex situ plant conservation program too (currently including 43 protected plant species, of which 17 are woody) [42,43].

**Figure 13:**
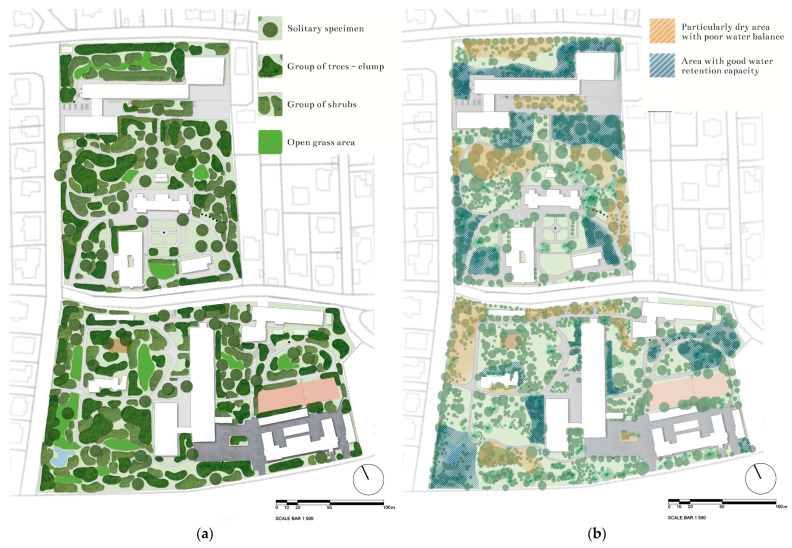
(**a**) The landscape characteristics and spatial structure; (**b**) the water retention capacity of the green area in the upper and lower gardens, Buda Arboretum (illustrations by Barnabás Tóth).

The arid, continental environment is characterized by an annual rainfall of only 600–620 mm, although in recent years it has been much lower at 450 mm. The garden is a so-called heat trap, as the surrounding urban fabric on the rapidly warming southern slope adds extra heating. The dry, less ventilated, and urban ‘smoke cloud’ over the university garden poses a long-term challenge in sustaining sensitive plants in wetter habitats. The poor to good water balance refers to previous local climate and ecological studies and observations (Figure 13) [35,39,42,44]. This heat-trap situation and the overall climate change, together with urban heat pollution, increase the environmental stress on the garden. Hence, the water-demanding species planted in the 20th century slowly died out; many other individuals became extinct, while drought-tolerant taxa from less water-sensitive or Mediterranean areas took over the dominance.

The arboretum is generally maintained on an extensive or semi-intensive level. Only three permanent gardeners work at the campus, while horticulture and landscape architecture students, and even external contractors, take part in the maintenance. During the COVID-19 pandemic, the students’ maintenance practices were prohibited due to the required exclusionary rules [45]. The arboretum was closed to visitors and residents looking for recreation and peace within the natural landscape character. On the other hand, there is no irrigation in most parts of the garden. Regular irrigation is only possible in certain priority areas, mostly in areas planted with intensive herbaceous vegetation.

### 4.2. Field Sampling and Data Collection

The study and its site analysis focus on the spontaneously reproducing species, including invasive and native taxa in the institutional green area, the Buda Arboretum. For the site analysis, directed in the summer of 2021, we divided the 7.5 hectare-large area into 22 sub-areas. Prior to the study, we verified the whole planting and all species in the maps, plans, plant lists, and other documents of previous field surveys [39]. The site analysis focused on fast and easy spreading species published in plant lists adapted to the local conditions and the national invasive species lists [22,27]. Adult specimens of all the species included in the study are present in the garden. Because of previous maintenance, there were no reference data for seedlings (spontaneously spreading specimens), only the COVID-19 pandemic restrictions and the lack of regular maintenance provided the unique situation in the almost abandoned arboretum worth this detailed study.

During the field survey, we recorded all spontaneously occurring woody species with a height of 20 cm. Species with vegetative reproducing, such as spreading rhizomes, stems, or root systems, received special concern to define their total occupied area; we separately checked the species threatened by adjacent, proposed, and established invasive plants to monitor the occupancy and the impact on the threatened species. The results were recorded in the arboretum map to define the ‘infested’ areas and identify taxa that may be of concern for maintenance in urban applications. Based on the number of individuals recorded, five categories of spontaneously occurring species were identified:

Category I—Intensively spreading taxa (> 100);
Category II—Spreading taxa (number of individuals 50–99);
Category III—Weakly spreading, and taxa with a low distribution (10–49);
Category IV—Just emerging (rare) (number of individuals 1–9);
Category V—Colonization species (species that reproduce only vegetatively and form large colonies)—not included in statistical evaluations.

### 4.3. Data Analysis

To reveal the possible differences or similarities in the data structure, a PCoA (Principal Coordinates Analysis) helped organizing the collected data along the properties of individuals/specimens (native or non-native spontaneously spreading trees). We used binary (presence–absence) and quantitative (number of individuals) data in the analysis. In PCoA, the distance matrix of objects was searched for a coordinate system where the original distances could be preserved, so the first few axes usually gave a reasonably good representation of the distances [46]. For data processing, the Euclidean distance resemblance matrix offered the proper way. For computation, we used the SYN-TAX 2000 program package [47]. Several statistical analyses performed in the survey used the MS Excel 2016 software: we made a regression analysis for the plot-by-plot assessment to define the relationship between green space and spontaneously spreading individuals and the correlation between spontaneously spreading and established individuals per plot. The diversity of the samples (‘parcels’) appeared in the number of taxa (species). The metrics used the PAST 4.12 software package [48]. Non-parametric tests (Mann–Whitney U test and the Kruskal–Wallis test) helped to compare the diversities. The dependent variable was the species number, and the independent variable was plots (1 to 22).

## 5. Conclusions

Botanical gardens and arboretums are unique plant societies of taxa from various remote countries, regions, and geographical areas; however, these plant collections can adapt to different climate circumstances and the separation from the original ecosystem and associations or the artificial plantation according to various compositional situations. Even the power balance among the newly assembled plant groups can be challenging for many species. Observing, evaluating, and predicting all these aspects of arboretums is a fundamental professional task within horticultural science [49,50,51].

The Buda Arboretum has a diverse, species-rich collection, with numerous artificial plant compositions that are, in many cases, based on taxonomic or plant geographical presentation ideas. There are many species in the garden that required more effort to conserve a few decades ago. These species had been planted in sheltered areas, for example, in front of retaining walls, to create a favorable microclimate. One example is the wax-leaved Privilege (*Ligustrum lucidum* W.T. Aiton), which has already reached a height of more than 10 m in its original planting site, and has spontaneously appeared in several parts of the garden due to climate change. Even though it spreads in this garden, we have the reports of Vera Csapody (an amateur botanist and famous illustrator for her plant drawings) of the Plant-friendly Company [52], stating that it froze in several other gardens. Primark and his colleague came to a similar conclusion: changes in global and local urban climates make it easier for previously sensitive species to survive the cold winters, and they can even become invasive [8]. Metropolitan heat islands may provide shelter and incubation opportunities for many warmth-loving, drought-tolerant newcomers [50,51], which, in the worst case scenario, escape from the city and expand in natural habitats.

Among the species in the garden, some are ideal for urban planting. These include the groundhog (*Baccharis halimifolia* Moench), which could be an excellent salt-tolerant plant, and so it may enrich a poor urban plant community; however, it is a potential invasive taxon in disturbed and saline areas [8,53,54,55]. We did not detect the groundhog spreading in the Buda Arboretum; however, it has produced viable seeds in other gardens, so an invasive behavior in our country cannot be excluded [56].

A potentially large number of species can ‘escape’ as a relevant consequence of a diverse collection garden with low maintenance; among the potential escapers, we can find a large number of native taxa. For example, the native maple (*Acer platanoides* L.) causes spreading problems in the Buda Arboretum or other collection gardens and urban open spaces in Hungary.

The land use variety and the fragmented space structure of the arboretum (Ménesi Street and various institutional buildings) affect the ecological conditions of the garden in terms of spontaneous species. On the one hand, buildings and built infrastructure create a barrier for dispersal, and this is the case of Ménesi Street. However, the buildings, as the hostel/school complex, have a light fragmentation effect; in this case, the different intensities of management of the two garden parts are responsible for taxa invasion problems.

Based on the experiences observed and collected in the garden, non-native species introduced into the urban environment as built-in elements can increase the aesthetic and recreational benefits [15] and have a positive ecological impact on urban green spaces [57]. However, ‘escaped’ species present significant ecologic and economic challenges [58,59]. Invasive plants can cause a decrease in biodiversity [15], a decline in the resilience of the ecosystem to disturbance, and degradation of the ecosystem [11]. In a world without borders, few, if any, areas remain sheltered from these immigrations [60]. The manifestation of invasive behavior depends not only on the species’ biological characteristics but also on random processes [61], while conscious human dispersal and selection activities contribute to the process [60]. The introduction of invasive or potentially invasive taxa by botanical gardens or the cultivation in nurseries can also trigger an intentional invasion [62], so collection gardens have a massive responsibility for plant use proposals in the future.

These research findings raise awareness of maintenance quality, and the justification of plants to avoid the spread of species listed as potentially invasive species; furthermore, this research draws attention to native species that require high maintenance efforts in gardens and large urban green spaces.

It is very important for botanical gardens to broaden their plant monitoring to respond to climate change issues, not only for the purposes of ornamental horticulture, but also for improving the maintenance of green spaces, and species conservation together with genetic resource communities [7]. The cooperation with economic actors such as nurseries should be improved to arrive at safe and sustainable horticultural production. Closer cooperation is essential for creating a sustainable environment in the long term. Priority should be given to the proper evaluation and testing of plant introductions for commercialization; recognition that the introduction of new plants into the country is not the most important part of the program but only the first step; full consideration of invasive species control policies and due diligence in assessing the potential risks posed by new introductions; and cooperation and exchange of experience between gardens.

## Figures and Tables

**Table 1:** The context for quantifying spontaneously propagating taxa and individuals in Buda Arboretum.

Spontaneously Spreading Plants		
	Taxa	Individuals
Native	38	2836
Non-native	76	4186
Total	114	7022

**Table 2:** The woody taxa in the Buda Arboretum according to the Black List categories * (management list in dark grey, action list in medium grey, and warning list in white) based on Bartha 2020 [27].

Species in BA	Risks of Biodiversity				Additional Criteria		Bio-Eco Criteria					
	Interspecific Competition	Hybridization	Transfer Pathogens	Negative Effect on Ecosystem	Current Distribution	Emergency Measure	Occurs in Important Habitats	Reproduction Cap.	Spread Cap.	Current Spread History	Monopolization of Resources	Facilitation of Climate Change
*Acer negundo*	yes	no	questionable	yes	large-scale		yes	high	high	expansive	yes	unknown
*Ailanthus altissima*	yes	no	no	yes	large-scale		yes	high	high	expansive	yes	yes
*Amorpha fruticosa*	yes	no	questionable	yes	large-scale		yes	high	high	expansive	yes	yes
*Celtis occidentalis*	yes	no	no	yes	large-scale		yes	high	high	expansive	yes	unknown
*Elaeagus angustifolia*	probable	no	no	yes	large-scale		yes	high	high	expansive	no	yes
*Fraxinus pennsylvanica*	yes	no	questionable	yes	large-scale		yes	high	high	expansive	yes	unknown
*Lycium barbarum*	yes	no	no	yes	large-scale		yes	high	low	stable	yes	unknown
*Parthenocissus inserta*	yes	no	no	yes	large-scale		yes	high	high	expansive	yes	unknown
*Prunus serotina*	yes	no	no	yes	large-scale		yes	high	high	expansive	yes	unknown
*Robinia pseudoacacia*	yes	no	questionable	yes	large-scale		yes	high	high	expansive	yes	yes
*Syringa vulgaris*	yes	no	probable	yes	large-scale		yes	high	high	expansive	yes	yes
*Ulmus pumila*	yes	yes	yes	probable	large-scale		no	high	high	expansive	unknown	yes
*Vitis vulpina*	yes	yes	yes	yes	large-scale		yes	high	high	expansive	yes	unknown
*Elaeagnus commutata*	questionable	no	no	yes	small-scale	available	unknown	high	high	unknown	yes	yes
*Hedera crebrescens*	probable	probable	questionable	yes	small-scale	available	no	high	high	expansive	yes	unknown
*Ptelea trifoliata*	questionable	no	no	yes	small-scale	available	yes	high	high	expansive	unknown	unknown
*Akebia quinata*	probable	no	no	yes	absent	available	yes	high	high	unknown	yes	yes
*Baccaris halimifolia*	yes	no	no	questionable	absent	available	yes	high	high	expansive	unknown	yes
*Eucalyptus* sp.	probable	no	no	yes	absent	available	no	high	high	expansive	yes	yes
*Ligustrum sinense*	probable	no	questionable	yes	absent	available	unknown	high	high	expansive	unknown	yes
*Pinus pinaster*	yes	no	probable	yes	absent	available	yes	high	high	expansive	yes	yes
*Toona sinensis*	yes	no	probable	probable	absent	unknown	unknown	high	high	unknown	yes	yes

* The species in the management list (dark grey) are already in the early stages of invasion, but the means to control them are unknown or they occur over a large area. The species in the action list (light grey) are also in the early stages of invasion but live in a small space and have the means to eradicate them. The warning list (white lines) is a collection of species considered to be flood species in areas with similar endowments, but they may not settle in the near future.

**Table 3:** The woody taxa in the Buda Arboretum according to the Grey List categories and their spontaneous appearance in urban environments.

Taxa in BA	Operative Group	Watch List	Spontaneous Appearance in Urban Environments
	For Natural Habitats		
*Acer pseudoplatanus* cv. Atropurpureum	x		x
*Acer opalus*		x	
*Broussonetia papyrifera*		x	x
*Buddleja davidii*	x		x
*Celtis australis*		x	x
*Cotoneaster divaricatus*		x	
*Cotoneaster horizontalis*		x	
*Cytisus scoparius*		x	
*Diospyros lotus*		x	
*Euonymus fortunei*		x	x
*Fallopia baldschuanica*		x	x
*Gleditsia triacanthos*		x	x
*Juglans nigra*		x	x
*Koelreuteria paniculata*		x	x
*Lonicera fragrantissima*		x	
*Lonicera × purpusii*		x	
*Lonicera standishii*		x	
*Mahonia aquifolium*	x		x
*Mahonia repens*	x		
*Morus alba*	x		x
*Parthenocissus quinquefolia*	x		x
*Paulownia tomentosa*	x		x
*Phyllostachys viridiglaucescens*	x		x
*Pinus nigra*	x		x
*Populus* × *euramericana*	x		x
*Prunus cerasus*	x		x
*Prunus mahaleb*	x		x
*Prunus cerasifera*	x		x
*Pterocarya fraxinifolia*		x	
*Rhus typhina*		x	x
*Robinia viscosa*		x	
*Rosa rugosa*		x	x
*Rubus phoenicolasius*		x	
*Yucca filamentosa*			x

**Table 4:** Correlation coefficients (r [−1, 1]) between spontaneously emerging/spreading and established individuals per plot (I–XXII) (bold: strong positive correlation r ≥ 0.7; species list is in alphabetical order).

**Non-Native/Non-Indigenous** **Intensively spreading taxa (> 100)** **Category I**	*Ailanthus altissima*	0.24
	*Celtis occidentalis*	0.16
	*Cotoneaster* spp.	0.59
	***Diospyros* spp.**	**0.75**
	*Koelreuteria paniculata*	0.40
	*Mahonia* spp.	0.11
	*Morus alba*	0.02
	*Parthenocissus* spp.	0.13
	***Prunus cerasifera***	**0.76**
	*Robinia* spp.	0.34
**Non-Native/Non-Indigenous** **Spreading taxa (number of individuals (50–99)** **Category II**	*Acer negundo*	0.56
	*Crataegus* spp.	0.48
	***Fraxinus* spp.**	**0.80**
	*Ligustrum* spp. (evergreen)	0.17
	*Ligustrum ovalifolium*	0.43
	*Smilax* spp.	0.39
	*Viburnum* spp.	0.18
**Non-Native/Non-Indigenous** **Weakly spreading, taxa with a low distribution (10–49)** **Category III**	*Aesculus hippocastanum*	0.12
	*Berberis julianae*	0.14
	*Cercis siliquastrum*	0.31
	*Cladrastris kentukea*	0.58
	*Cotoneaster multiflorus*	0.01
	***Fontanesia phillyreoides***	**0.99**
	***Gymnocladus dioicus***	**1.00**
	***Lonicera japonica***	**0.83**
	*Parrotia persica*	0.52
	*Populus* spp.	0.40
	*Prunus* spp. (mahaleb)	0.28
	*Sophora japonica*	0.01
	*Symphoricarpos* spp.	0.24
	*Tetradium daniellii*	0.44
	***Toona sinensis***	**1.00**
	*Vitis* spp.	0.46
	***Wisteria* spp.**	**0.92**
**Native/Indigenous**	*Acer campestre*	0.44
**Category I–III**	*Acer platanoides*	0.55
	***Acer pseudoplatanus***	**0.75**
	***Acer tataricum***	**0.73**
	*Corylus avellana*	0.28
	*Crataegus* spp.	0.48
	*Fraxinus excelsior*	0.27
	*Fraxinus ornus*	0.16
	*Ligustrum vulgare*	0.07
	*Lonicera tatarica*	0.12
	*Prunus avium*	0.50
	*Prunus padus*	0.15
	*Quercus* ssp. (deciduous)	0.31
	*Sambucus nigra*	0.03
	*Taxus baccata*	0.33
	*Tilia* spp.	0.52
	*Tilia tomentosa*	0.42
	***Ulmus* spp.**	**0.72**
	*Viburnum* spp.	0.18

**Table 5:** Rate of spontaneously appearing and planted individuals in Buda Arboretum.

Ranking	Species	Spontaneous Number of Individuals	Planted Number of Individuals	Rate
1	*Celtis occidentalis*	561	3	187
2	*Ailanthus altissima*	432	3	144
3	*Koelreuteria paniculata*	258	5	52
4	*Acer campestre*	216	5	43
5	*Diospyrus lotus*	157	4	39
6	*Acer platanoides*	647	18	36
7	*Morus alba*	108	3	36
8	*Acer pseudoplatanus*	193	6	32
9	*Prunus cerasifera*	337	20	17
10	*Acer negundo*	52	4	13

## Data Availability

Not applicable.
